# Acromegaly: Role of Surgery in the Therapeutic Armamentarium

**DOI:** 10.1155/2012/306094

**Published:** 2012-11-12

**Authors:** Gerardo Guinto, Miguel Abdo, Erick Zepeda, Norma Aréchiga, Moisés Mercado

**Affiliations:** ^1^Department of Neurosurgery, Hospital de Especialidades, Centro Médico Nacional Siglo XXI, 06720 Mexico City, DF, Mexico; ^2^Centro Neurológico ABC, 05300 Mexico City, DF, Mexico; ^3^Department of Neurology, Hospital de Especialidades, Centro Médico Nacional Siglo XXI, 06720 Mexico City, DF, Mexico; ^4^Department of Endocrinology, Hospital de Especialidades, Centro Médico Nacional Siglo XXI, 06720 Mexico City, DF, Mexico

## Abstract

Acromegaly is a complex disease that requires the intervention of a multidisciplinary team. The most frequent clinical manifestations are growing of distal parts of the body and some areas of the face. Patients may also present arterial hypertension, diabetes mellitus, colonic polyps, cardiomegaly, neurological and endocrine changes secondary to the presence of a GH-secreting tumor in pituitary or extrapituitary origin, or eutopic hypothalamic GHRH hypersecretion and peripheral GHRH hypersecretion. Surgery is the first treatment used for most patients, regardless of the cause. In the great majority of cases, pituitary tumor can be removed through a transsphenoidal approach. Craniotomy is reserved for those cases with giant tumors, particularly when they grow toward the middle or posterior cranial fossa. Best surgical results are obtained when the tumor is confined into the sella turcica or if it has a regular suprasellar extension. When the disease cannot be controlled with surgery, medical treatment is indicated. Somatostatin analogues are included as the first line of medication, followed by dopamine agonist and growth hormone receptors antagonists. Radiation therapy can be also indicated in two main forms for residual tumor with medically refractory patients: radiosurgery for small tumors or fractionated stereotactic radiotherapy for larger ones.

## 1. Introduction

Acromegaly is a chronic, progressive, and multisystem disease characterized by the hypersecretion of growth hormone (GH), which in turn generates an excessive amount of IGF-1 (insulin-like growth factor-1), which mediates most of the effects of GH [1, 2]. In >95% of cases, the cause is attributed to a somatotroph pituitary adenoma. It has been demonstrated that up to 40% of these adenomas secrete other hormones, especially prolactin and thyrotropin, which may or may not be clinically evident [1–3]. The remaining 5% of cases are due to an ectopic secretion of the GH-releasing hormone (GHRH) by a hypothalamic tumor, carcinoid tumor (of the lung or gastrointestinal tract), or pancreatic islet tumors. Small-cell lung carcinomas and pheochromocytomas may, on rare occasions, also produce this hormone [2, 3].

## 2. Clinical Remarks

The prevalence of acromegaly is estimated to be 40–70 cases per million inhabitants with an incidence of 3–5 new cases per million inhabitants per year. The classic clinical manifestations of the disease focus on the enlargement of hands, feet, and facial bones (acral growth) including the prominence of supraorbital ridges and growth of the nose and jaw, causing prognathism and separated teeth [2, 4, 5]. There are also other visceral or systemic manifestations such as hyperhidrosis, heat intolerance, arthritis, fatigue, cardiomegaly, macroglossia, atherosclerosis, systemic arterial hypertension, acne, sleep apnea, and insulin resistance. In addition, patients have a 2.4 to 2.7 times increase in the prevalence of colon polyps when compared with the general population which, in turn, increases the risk of colon cancer.

Because pituitary adenoma is the most common cause of this disease, there are usually symptoms attributed to tumor growth in the sella turcica such as headache and signs of compression of the visual pathway that causes a bitemporal hemianopsia [6–9]. Also, the progressive expansion of the tumor may cause a decrease or loss of function of the pituitary gland, producing varying degrees of hypopituitarism. Of all primary tumors of the pituitary, between 15, and 20% are GH-secretors. When untreated, acromegalic patients have a high morbidity and mortality because patients with the disease have a decreased life expectancy by at least 10 years when compared with the general population [1, 5, 10]. Causes of death are particularly due to cardiovascular and respiratory problems and to the increased risk of neoplasms.

## 3. Diagnosis

Unfortunately, symptoms of acromegaly have a subtle and insidious beginning, for which reason the diagnosis is often delayed by an average of 7-8 years. Although clinical findings are characteristic, over production of GH should be documented.

For GH determination, it should be considered that the secretion is pulsatile; therefore, random measurements are not appropriate for diagnosis [11, 12]. As IGF-1 has a long half-life of 18 to 20 hours, IGF-1 level is recommended as the best initial screening test for all patients suspected of having acromegaly. In addition to reflecting GH secretion at 24 h, it requires a single sample and does not generate patient discomfort. However, an elevated IGF-1 adjusted for age and gender should be repeated after 2 or 3 weeks because levels can vary up to 30% [12]. Although this test may be sufficient to establish the diagnosis in some centers, if a greater certainty is desired, GH measurement is further recommended in response to an oral glucose tolerance test (OGTT) (75 g) [13–16]. Under normal conditions, with this carbohydrate load, it is expected that GH production will be suppressed to levels <1 ng/mL, although recent “ultrasensitive” assays for GH detection estimate that normal suppression should be <0.4 ng/mL [5, 10, 11, 14, 17, 18].

The positive results of these tests provide only a state of GH hypersecretion that, although in most cases is due to the presence of a pituitary adenoma, it is not always possible to ensure this, even if there is a radiological study showing the presence of a mass in the sellar region [3, 4]. The remote possibility of the presence of an extrapituitary GHRH-producing tumor must be considered because these lesions, in addition to increasing GH levels, can cause a somatotropic hyperplasia and suggest the presence of a pituitary adenoma on imaging studies. To avoid this confusion, measurement of GHRH level by radioimmunoassay must be performed in doubtful cases.

Endocrine evaluation must include an analysis of all hypothalamic pituitary axes in order to detect possible associated deficits that will need to be corrected, particularly if the patient will undergo surgical management. Once there is a clinical and paraclinical suspicion of a pituitary tumor, the next step is to submit the patient to an imaging study. Undoubtedly, magnetic resonance imaging (MRI) is the most useful study because it not only detects the presence of a very small tumor, but also provides all the details of adjacent structures and their relations to the tumor, information necessary to determine the best therapeutic decision [6–8].

When the tumor is <4 mm, its identification with standard MRI is very difficult [3]. Currently there are special techniques such as the spoiled-gradient recalled (SPGR), which provides high-definition images of the soft tissues that can detect these small tumors. Besides, half-dose dynamic gadolinium-enhanced MRI with simultaneous acquisition of coronal and sagittal planes is also very useful for the detection of these small lesions [19]. In cases of tumors with suprasellar growth, it is also necessary to perform a complete ophthalmological evaluation to determine and define a possible visual deficit. This study will also serve as a reference for assessing the effectiveness of treatment and to provide appropriate clinical followup in the medium- and long-term period [9].

## 4. Treatment

Once the diagnosis of a GH-secreting adenoma is made, it should be kept in mind that acromegaly is a multisystem disease. Therefore, therapy should cover all aspects of it, especially neurological, oncological, and endocrinological, which implies the involvement of a multidisciplinary team.

Treatment goals are as follows:

1. removal of the mass effect of the adenoma and restoration of neurological function,
2. normalization of GH secretion (biochemical remission),
3. preservation of pituitary function,
4. reduction of the risk of cancer and/or biochemical recurrence,
5. treatment of related complications.

Surgical resection is the initial treatment of choice for the vast majority of patients with acromegaly [4, 5, 14, 15]. Efficacy of surgery depends on several factors including the size of the tumor, its growth pattern, and preoperative levels of GH. However, the most important factor is undoubtedly the experience of the surgical team.

## 5. Surgical Indications and Contraindications

A patient with the clinical syndrome of acromegaly that presents a pituitary tumor associated with biochemical evidence suggestive of GH hypersecretion and absence of serious concomitant diseases is the main indication for surgery. The appropriate timing for surgery will be decided by mainly taking into account the tumor growth pattern. In most patients, surgical management is not considered an emergency and can be programmed at an optimal time as determined by the multidisciplinary team. The following cases are considered to be indications for an emergency surgery: rapidly progressive visual deficit, clinical or radiological evidence of tumor apoplexy, or the presence of cerebrospinal (CSF) leak (which is rare in acromegaly) [9, 16, 17]. These events are seen more frequently in macroadenomas (>1 cm) than in microadenomas (≤1 cm).

Another indication for surgery is in cases where, for some reason, it was decided to initiate medical treatment with somatostatin analogues and/or dopaminergic agonists, but which are resistant or intolerant, especially in the presence of a morbid type of acromegaly.

At present, due to the advanced development of microsurgical techniques, contraindications for surgery are really few and relative. Most patients of advanced age and/or with related concomitant diseases may be operated, especially if removal of the tumor can be done through transsphenoidal route. Only those cases with current clinical problems and presenting large macroadenomas where performing a craniotomy is required are those in whom surgical management is contraindicated. Also, the presence of an infectious process, coagulation problems, or metabolic imbalance must be resolved prior to surgery.

## 6. Preoperative Management

All problems related to acromegaly such as hypertension, ischemic heart disease, cardiomyopathy, peripheral vascular disease, thyroid disorders, and diabetes mellitus should be managed before, during, and after surgery. Special mention must be made in relation to the growth of the jaw and tongue as well as dental malocclusion and poor temporomandibular joint function because these problems may complicate airway management during induction of anesthesia and especially during endotracheal intubation.

In these cases, medical management should be considered before surgery, which can reduce the appearance of these technical difficulties that increase surgical risk [8, 18]. In addition, there are case reports of large or invasive adenomas in which the administration of somatostatin analogues caused a reduction in tumor size and a decrease in its consistency, facilitating surgical removal [4, 8, 9, 18].

## 7. Surgery

There are two approaches to surgically remove somatotroph adenomas: transsphenoidal (TS) and transcranial (TCr) [9]. Currently >90% of these tumors are approached by the TS route because it offers a better balance between the degree of tumor resection and the risk of postoperative complications [8]. The TS approach has two varieties: microsurgical and endoscopic. TS microsurgical approach is performed under the microscope and can be subdivided into three types: sublabial (when performed through an incision parallel to the gingival-labial sulcus), endonasal submucosal (when performed through an incision in the mucocutaneous junction of the nasal septum), and direct endonasal (when no submucosal dissection is performed, but direct access to the anterior wall of the sphenoid sinus is gained through one of the nostrils). The specific choice of each of these variants predominantly depends on the surgeon's experience and preference. However, in larger tumors, particularly those with significant lateral or suprasellar growth, the sublabial approach is preferred because it allows a discretely wide rexposure than the other two routes. In small tumors there is a tendency to choose one of the endonasal approaches because these present greater patient comfort, considering that in most cases, postoperative nasal packing can be avoided.

The TS endoscopic approach has gained increasing popularity because it offers better illumination and a wider view of the surgical field. It is most useful in macroadenomas where there are portions of the tumor that are hidden from the surgeon's angle of vision. However, its main disadvantage is that it allows only a two-dimensional view, which causes loss of the sense of depth of the surgical field, a reason that a surgeon who has mastered the microsurgical technique must restart the learning curve in order to use the endoscopic approach. But a novel 3D visualization system for endoscopic endonasal transsphenoidal surgery has improved in depth perception, as it was appreciated by senior surgeons involved, and there was no increase in complications or operative time. This technology supports the feasibility and safety of this technology and becomes available to practicing transsphenoidal surgeons. It has the potential to correct the limitations of traditional 2D endoscopic technology.

The TCr approach has multiple modalities, the most frequent of which are mentioned: pterional or frontotemporal, unilateral subfrontal, bilateral subfrontal, and interhemispheric subfrontal. In all of these approaches there is a natural tendency to make even smaller approaches in what is termed minimally invasive surgery.

## 8. Selection of Approach

**Figure 1:**
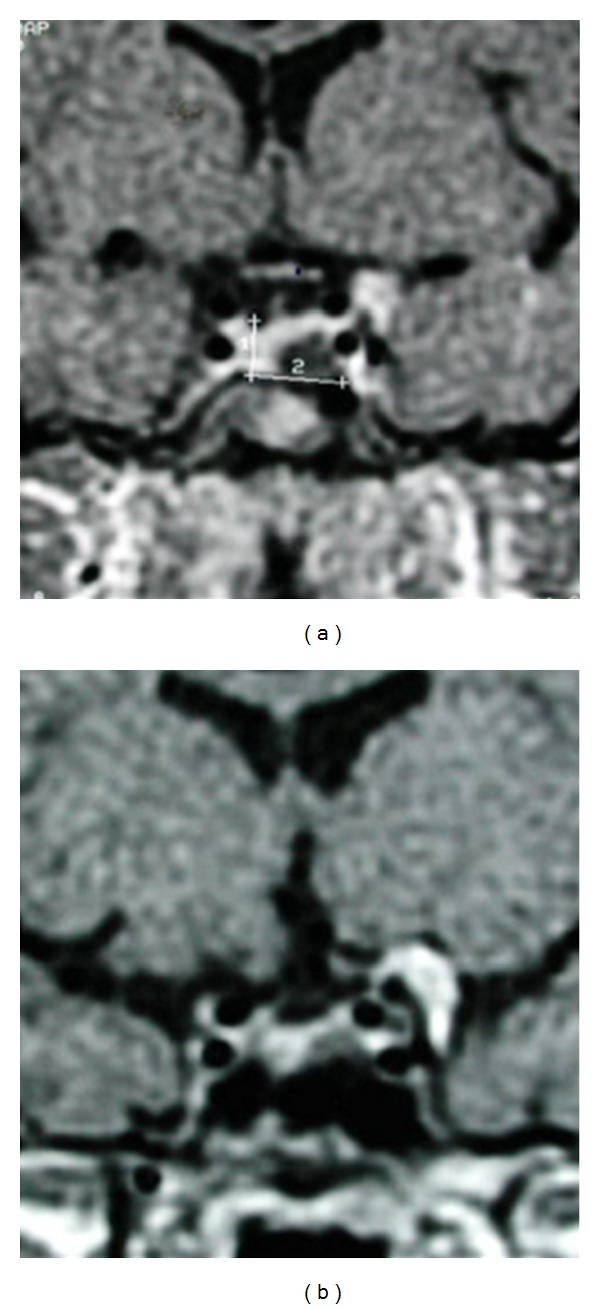
Ideal tumor for surgery. (a) Contrast enhanced MRI in coronal view showing a small tumor (line number 2) located on the left part of the sella turcica. Pituitary gland (line number 1) appears slightly displaced. (b) Postoperative result. In this case, a clinical and biochemical remission could be obtained.

**Figure 2:**
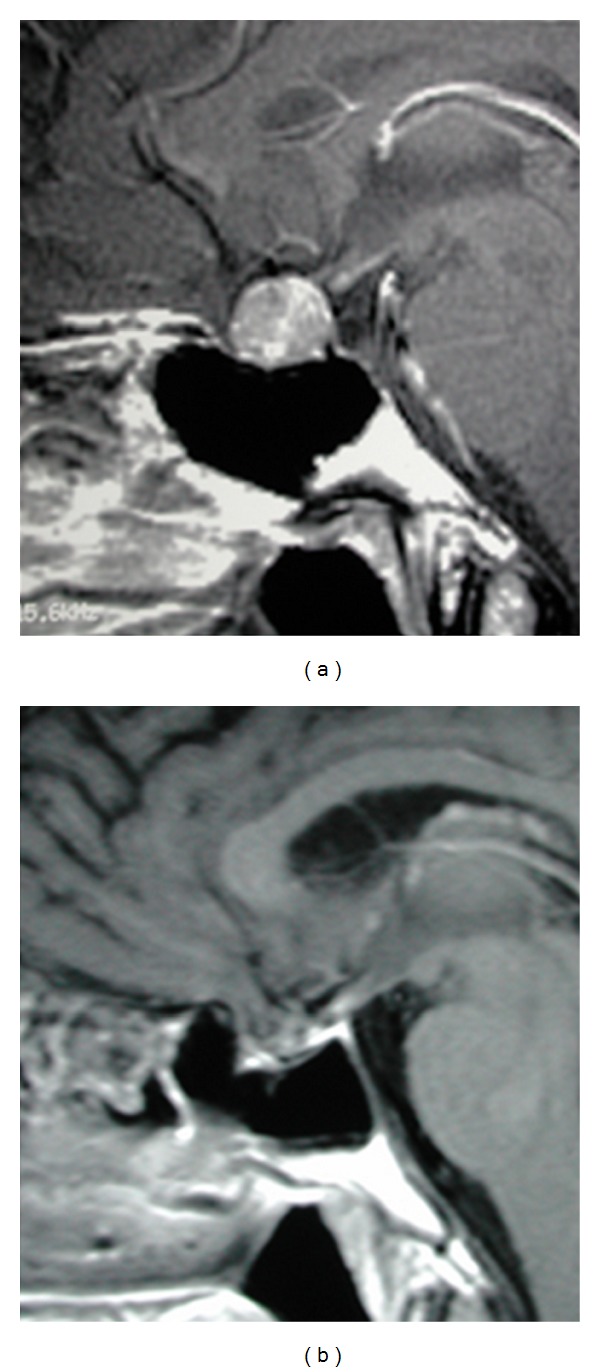
Contrast enhanced MRI sagittal view showing an intrasellar pituitary adenoma (a) that could be completely removed with surgery (b). Biochemical remission was also gotten.

**Figure 3:**
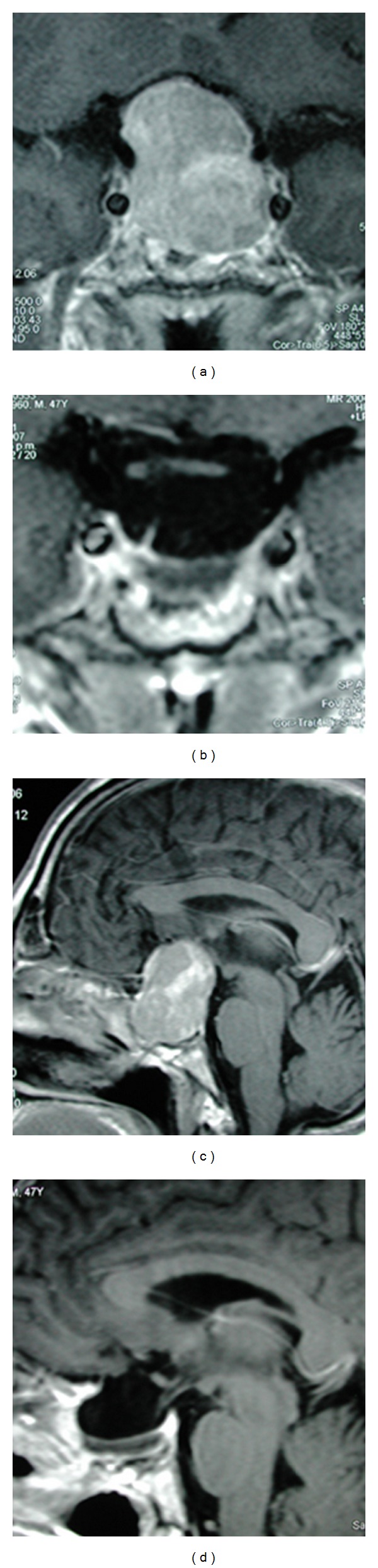
Large pituitary adenoma that presents a uniform suprasellar growth. Macroscopic total removal of this tumor could be obtained through a TS approach. (a, c) Preoperative imaging (upper coronal and lower sagittal). (b, d) Postoperative result in the same projections.

**Figure 4:**
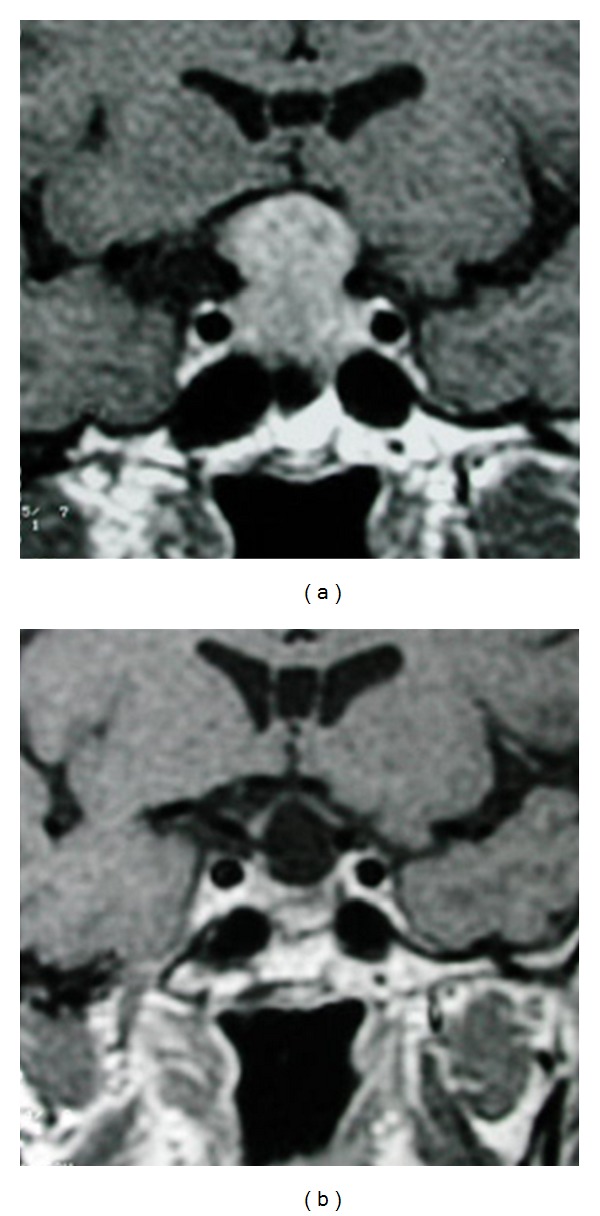
Typical “snowman” or “dumbbell” shape appearance of a pituitary adenoma causing acromegaly. In this case, even though the tumor was macroscopically removed, the patient was not cured with surgery and had to be medically treated. (a) Preoperative imaging and (b) postoperative result.

The ideal tumor that represents the highest cure rate with surgery is the intrasellar microadenoma, especially when it is lateralized and well localized in the imaging studies. In these cases, using the TS approach, a biochemical cure and preservation of the hypothalamic-pituitary axis can be achieved in the vast majority of patients (Figure 1). Intrasellar macroadenoma is the second in frequency where highly successful results can be achieved in terms of remission (Figure 2). The next group of tumors includes adenomas which present a uniform suprasellar growth (Figure 3). When this variety of tumor shows a central narrowness (caused by the diaphragm sellae), a morphological appearance of a “snowman” or “dumbbell” is acquired (Figure 4). In some of these lesions, the narrowness and tightness in the central portion of the tumor obstructs adequate visualization of the upper portions. A TS approach is performed as the first procedure, attempting to do the resection as wide as possible. According to the results evaluated in control imaging studies performed 6–8 weeks later, another surgery will be planned to remove the residual tumor, whether by the same approach if it descended to the sella turcica or via a TCr approach if it remains in the suprasellar region. In these cases, biochemical cure of acromegaly is virtually impossible with surgery alone, even if there is no evidence of residual tumor on control imaging studies.

**Figure 5:**
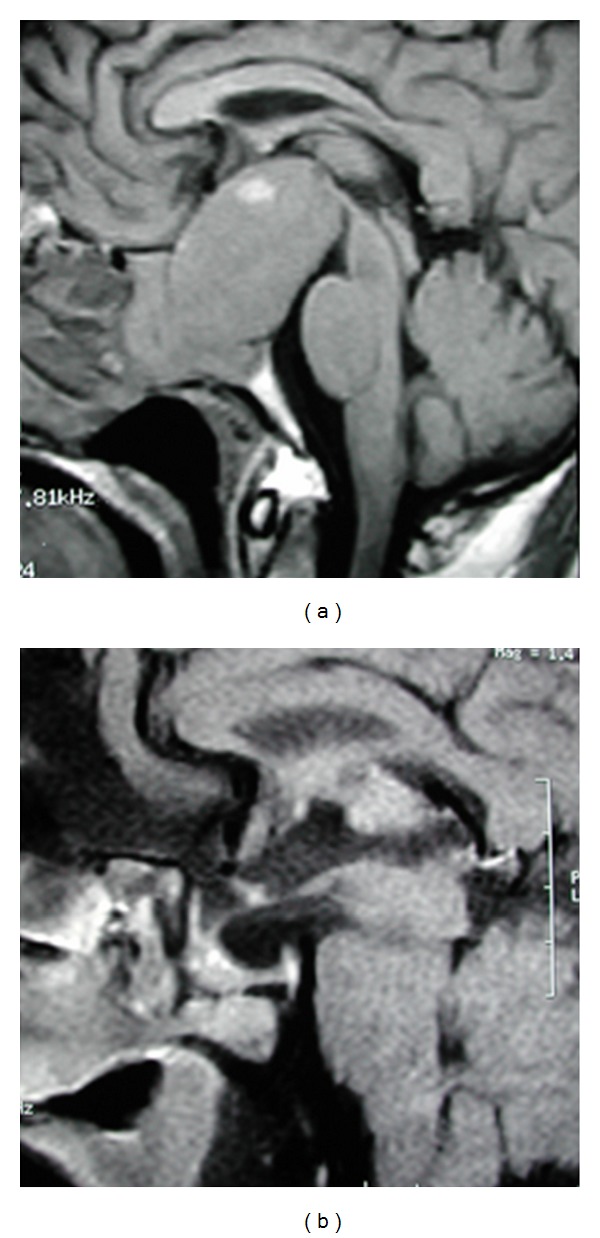
Enhanced MRI sagittal view that shows a giant pituitary adenoma reaching the third ventricle. Considering the uniform shape of the tumor, it could be removed through TS approach. (a) Preoperative view and (b) postoperative result.

**Figure 6:**
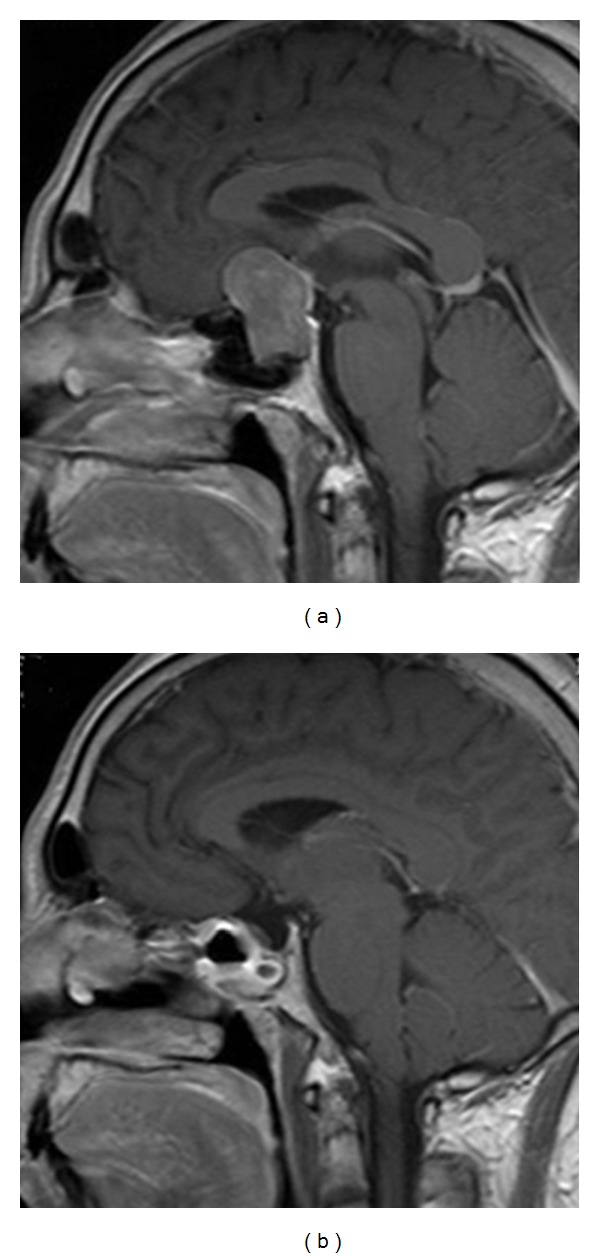
Enhanced MRI sagittal view. In this case, the tumor presented a small portion growing toward the anterior cranial floor (a). An extended TS approach was used and the tumor could be successfully taken out (b).

When the adenoma reaches the third ventricle but still presents a uniform shape, it can also be removed by a TS route (Figure 5). Tumors with a small portion growing toward the anterior floor may also be removed using the TS approach (Figure 6), whether with endoscopy or microsurgery in what is called extended TS approach. This involves removing part of the anterior floor of the skull base, to make tumor exposure wider.

The most frequent indications for the TCr approach is extrasellar tumor growth (predominantly lateral or posterior), and when the transsphenoidal approach has failed, but if it was performed by a skilled neurosurgeon.

## 9. Invasion to the Cavernous Sinus

One of the most controversial issues regarding the treatment of invasive pituitary adenoma is the invasion to the cavernous sinus (CS). The CS is a paired structure located on the sides of the sella turcica and is the site where the internal carotid artery, the oculomotor nerves, and the first or occasionally the second branch of the trigeminal nerve cross. This space is also filled with a capillary plexus and venous dilatations. In fact, the carotid artery (surrounded by the sympathetic plexus) and the sixth cranial nerve are strictly the only intracavernous structures because the third and fourth cranial nerves and branches of the fifth cross through its lateral wall.

**Figure 7:**
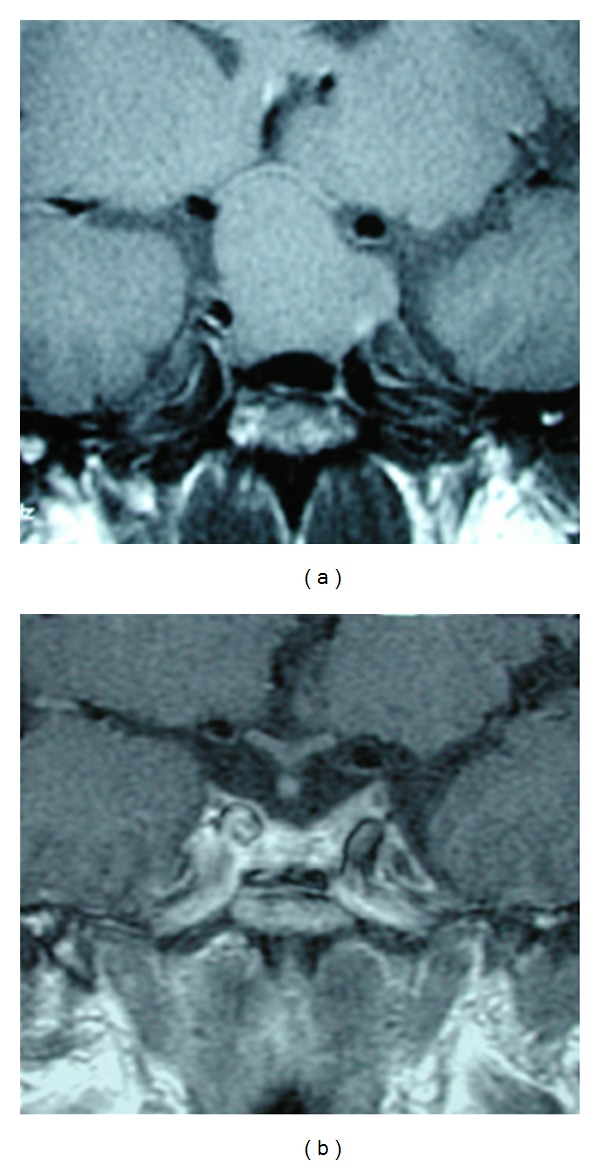
Enhanced MRI coronal view that shows a tumor which is only displacing (but not invading) the left CS (a). On postoperative control, imaging (b) is possible to observe that the whole tumor was macroscopically removed. TS approach was used because the CS was not really invaded.

**Figure 8:**
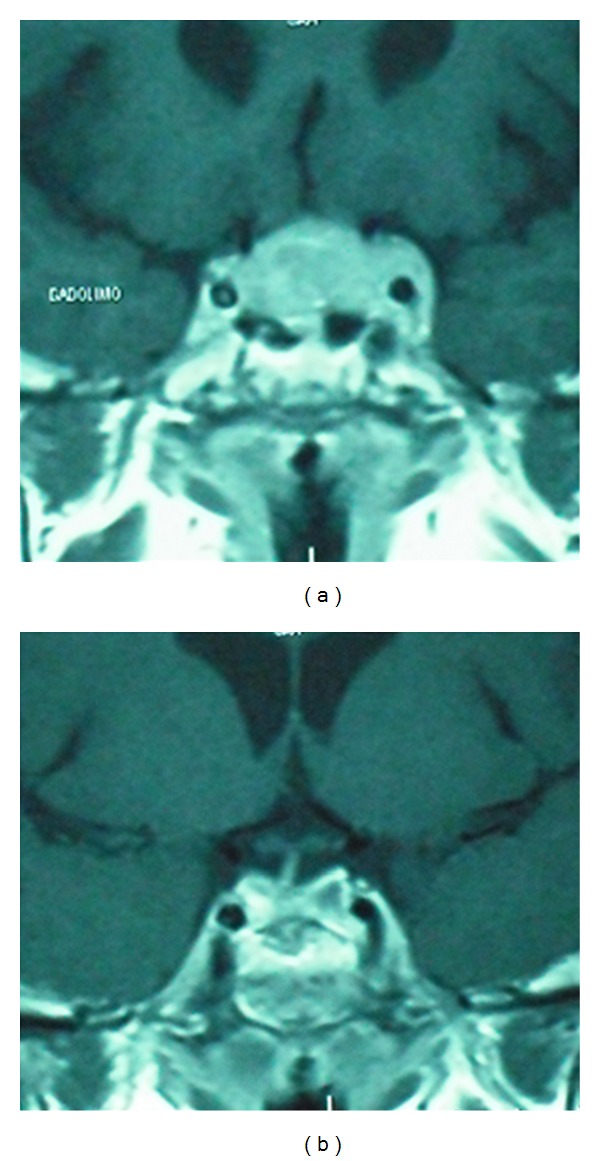
Enhanced MRI coronal view. In this case, the left CS was really invaded. Observe how the left carotid artery is completely surrounded by the tumor (a). Patient was operated on through a TS approach and fortunately, the tumor was soft and so, it could be removed, including the intracavernous portion (b). However, biochemical remission of the disease could not be obtained.

**Figure 9:**
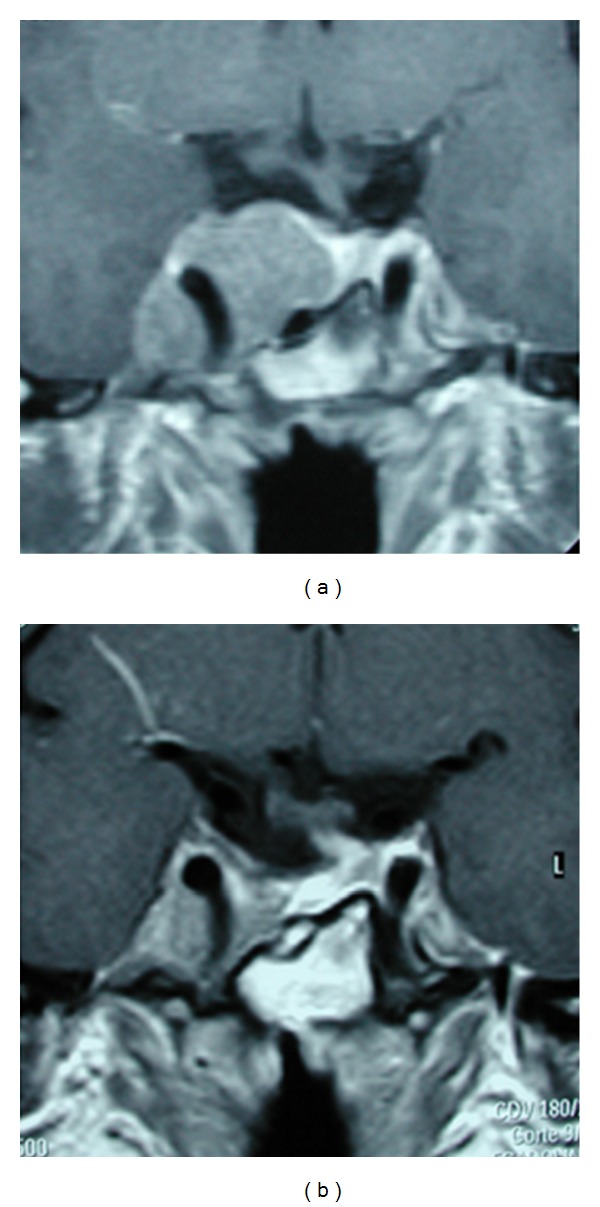
Another case of real invasion to the CS. Note how the right carotid artery is surrounded by the pituitary adenoma (a). Tumor presented a fibrous consistency and the intracavernous portion had to be left behind (b). Radiosurgery was applied as adjuvant treatment.

Only on rare occasions, pituitary adenomas present a real invasion to the CS because in most cases they cause only a displacement of this structure and can be easily removed through a TS approach (Figure 7). Considering that virtually any surgical approach to the CS provides a high risk of oculomotor or carotid injury, in acromegaly cases a direct approach to this area is almost never indicated. The TS approach continues to be the most selected route where the primary objective is to completely remove the entire sellar tumor component, if the cavernous portion is soft, in some cases it can also be removed through this route (Figure 8). However, if this portion is fibrous, it is preferable to leave the intracavernous part for subsequent management either with medical treatment and/or radiosurgery (Figure 9).

## 10. Surgical Results

After surgery, the majority of patients for whom biochemical remission is achieved and those in whom GH levels are significantly lower (but are not normalized) are expected to rapidly improve some of their symptoms. Headache, hyperhidrosis, tissue edema, and paresthesia improve almost immediately [20]. Hyperglycemia also responds favorably in the first weeks, but not hypertension because previously established changes in blood vessels and heart are usually not reversible [21]. Furthermore, acral growth regresses less often as a result of the treatment and generally continues for the remainder of the patient's life. However, when the disease is controlled, these growth changes are stopped, which improves the aesthetic perception and represents one of the positive aspects for patients, particularly females.

## 11. Definition of Remission and Control

During the past two decades, the concepts of remission or “cure” and control of the disease have changed [5, 10, 14]. Remission or “cure” of acromegaly is defined based on two criteria: a normal IGF-1 level adjusted for age and gender and a random GH level <1.0 ng/mL (using an ultrasensitive assay). Disease control is defined when there is a GH value <2.5 ng/mL [5]. If this is achieved, disease comorbidity is significantly reduced, especially mortality.

Biochemical regularization does not appear immediately after surgery so that testing is recommended 3–6 months after the procedure [14, 18]. Similarly, imaging studies to assess the presence of residual tumor are not done immediately, and it is also recommended that they be carried out between 2 and 3 months after surgery.

Patients considered to be in remission shall be followed biochemically (IGF-1 and GH post-OGTT) annually for 5 years [5, 10]. Performing serial MRI is not indicated unless there are data of new visual deficit or evidence of residual tumor in control studies.

If 3 months after surgery the IGF-1 adjusted for age and gender remains high and GH after OGTT is not adequately suppressed, it is considered that the patient is biochemically active and a decision must be made whether the patient is a candidate for a reoperation, other forms of drug therapy or radiation therapy. In patients with mild biochemical activity, that is, minor elevations in IGF-1 and/or suppression of GH <1 ng/mL but who did not reach the nadir seen in normal patients, treatment is not required unless there is evident clinical activity or presence of residual tumor on MRI. 

Finally, it is important to add that between 10% and 15% of patients show discordant biochemical data after surgery because they exhibit normalization of GH levels but IGF-1 remaining high. It is recommended that these patients be observed and reassessed in 6–8 months. If there is biochemical activity during the second evaluation, application of a new therapeutic option must be considered [22, 23].

**Figure 10:**
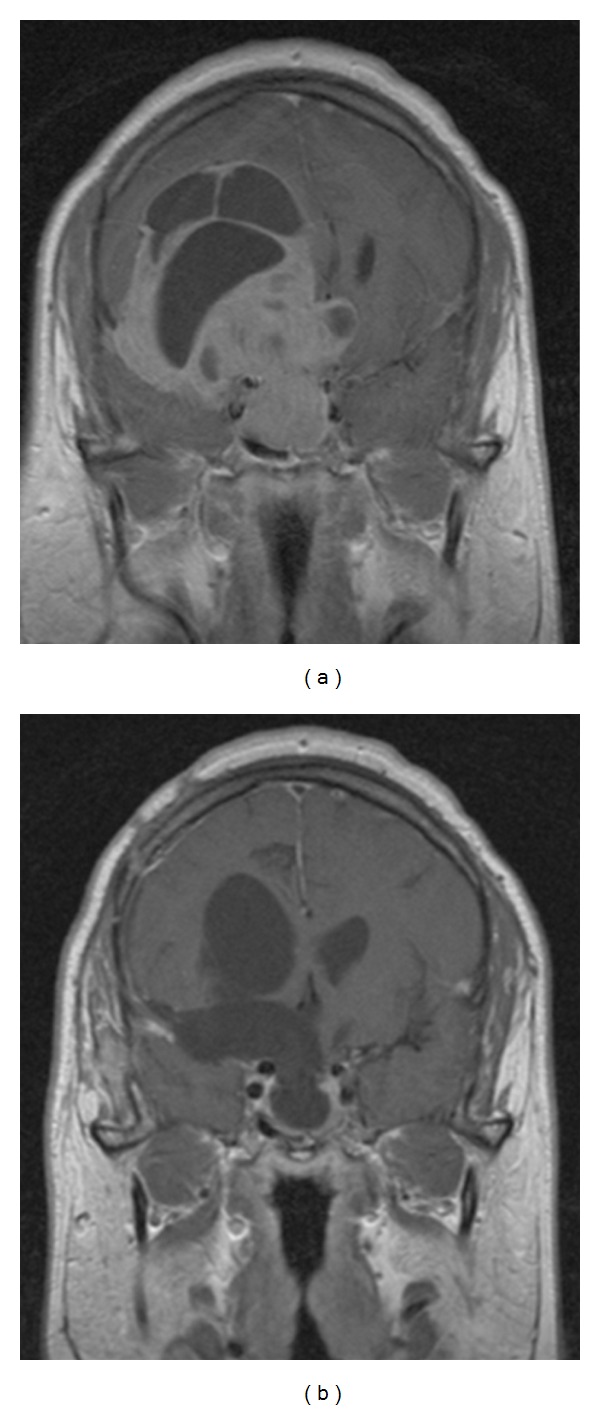
Enhanced MRI coronal view of a giant tumor growing toward the middle cranial fossa (a). A combined TCr approach was used in this case and even though the whole tumor could be removed (b), acromegaly persisted and the patient was treated with somatostatin analogues.

The main factors influencing surgical prognosis are size and growth pattern of the adenoma. In microadenomas, an experienced surgeon can achieve a rate of remission in between 66% and 90% of cases, whereas for macroadenomas this rate decreases to 40%–50% and falls further when there is extrasellar extension or CS invasion. Cure rates or remission are almost negligible when the TCr approach is used, which obviously is due to size and pattern of tumor growth than to the approach itself (Figure 10).

## 12. Recurrence

There are cases of actual recurrence in acromegaly, that is, those patients who met the criteria for remission but who present again the clinical and biochemical syndrome. However, cases of persistence are more frequent where it was not possible to achieve cure or remission with the first surgery. Both situations should be examined individually in order to decide whether the patient should be subjected to additional surgery or another treatment modality. However, it must be accepted that the chance for a cure with a second surgery is almost nil, even in cases of intrasellar adenomas. The best chance for remission or cure of the disease is during the first surgery, the reason why this procedure must be performed by a highly qualified surgical team [4, 16].

## 13. Complications

The spectrum of complications associated with surgery for GH-secreting adenomas are the same as those reported in surgeries of any type of adenoma. The most frequently reported complications when using the TS approach are inherent in the approach [6, 18] and include dental hypoesthesia or anesthesia, septal perforations, rhinitis, nasal crusting, hyposmia, or anosmia. These complications are more frequently mentioned in submucosal variants, especially sublabial. CSF leak is another complication referred to, particularly in centers without great experience. The best method to avoid this complication is to precisely identify the defect on the sellar diaphragm during surgery for the correct sealing and proper reconstruction of the floor of the sella turcica, preferably with the use of autologous bone. This reconstruction is more difficult to perform when using the endoscopic approach where it is recommended to seal the floor of the sella turcica with a pediculated flap of septal mucosa.

Undoubtedly, the most feared complication of any of the TS approaches is the tearing of the internal carotid artery because this represents the leading cause of severe morbidity and even mortality. In these cases it is impossible to obtain an adequate repair of the vascular defect during surgery and the surgeon is forced to only packing the site of bleeding with hemostatic material. After surgery, the patient must be submitted to an angiography and to attempt to repair the defect by using coils, stents, or detachable microballoons through an endovascular approach. If this is not immediately done, there is a high risk of formation of pseudoaneurysm or carotid-cavernous fistula. 

Other complications reported in the TS approach that place in jeopardy the life of the patients are hypothalamic damage and subarachnoid hemorrhage. These occur especially when the tumor has a significant suprasellar growth and the surgeon is forced to carry out traction maneuvers to remove the highest parts of the tumor. Visual system damage is another serious complication that has multiple causes, among which are direct injuries during the opening of the sellar floor (particularly when using the chisel), over traction of the suprasellar structures, overpacking during reconstruction, and the presence of a postoperative hematoma in the surgical bed. In almost all these cases, immediate reoperation is indicated to correct the problem. If not corrected within the first few hours, the visual deficit is usually permanent.

The most frequently reported complications for the TCr routes are related to lesions in the hypothalamic-pituitary axis. The rate of diabetes insipidus and panhypopituitarism are higher than in TS approaches [24, 25] and in many cases are permanent. Lesions to the hypothalamic control centers are also reported, but fortunately with a low frequency. In this situation, patients become lethargic and/or comatose after surgery. Finally, a group of general complications associated with any other cranial surgery must be added to TCr approaches, which include hematoma, infection, stroke, CSF leak, seizures, concussions, and cerebral edema.

## 14. Predictors of Surgical Results

There are several factors that influence surgical outcomes for acromegaly and can be classified as follows.

- Patient-related factors: presentation of acromegaly in patients <25 years of age is considered to be a poor prognostic factor [26]. Patients suffering from gigantism represent a well-recognized group of patients who are refractory to surgical treatment and cure is rare. The cause of this lack of response is unknown. Another factor that negatively influences the result is a history of previous surgery.
- Factors related to the tumor are as follows.
  1. Secretory activity: there is an inverse relationship between preoperative GH levels and postoperative result [27]. Patients with GH levels <50 ng/mL preoperatively have greater possibility of cure than patients with levels >50 ng/mL.
  2. Tumor size: microadenomas present better results than macroadenomas [4].
  3. Tumor growth pattern: in tumors without extrasellar extension there is a greater chance of achieving remission with surgery alone (60%–73%) as compared to those with extrasellar extension (0%–27%) [28].
  4. Tumor invasiveness: macroscopic as well as microscopic invasion of the dura mater or CS adversely affect surgical success.
  5. Histological appearance: tumors cosecretors of GH and prolactin have a poorer prognosis than tumors that secrete only GH.
- Factors related to the surgeon: these are the least objective ones because it is very difficult to define when a surgeon is properly qualified to perform these surgeries. It is generally accepted that to be considered a pituitary surgeon, the surgeon must perform at least 30 such procedures a year.

## 15. Medical Treatment

In general terms, 50% of patients undergoing surgery for acromegaly will achieve clinical and biochemical remission. The remaining patients require some form of adjuvant treatment [15, 29, 30]. However, there is a group of patients in whom medical treatment is considered as the best and/or only option. These include the following conditions: invasive macroadenomas with very little intrasellar component, patients with severe cardiopulmonary contraindications, absence of an expert pituitary neurosurgeon, and/or patient preference.

### 15.1. Management Options

The aim of medical treatment is to control the disease, reducing the risk of morbidity and mortality comparable to the general population [29–31].

#### 15.1.1. Somatostatin Analogues

Somatostatin is a protein molecule widely distributed throughout the body with a half-life of 2 min. Multiple functions have been described for somatostatin including inhibiting GH and TSH release, decreasing secretion and motility of the stomach and duodenum, and decreasing secretion of glucagon and insulin (among others) [32].

Somatostatin exerts its physiological effects by stimulating G protein-coupled receptors as second messengers. To date, five types are known and the inhibition of GH secretion is preferentially mediated by receptors 2 and 5 [31]. GH-producing pituitary tumors also express these types of receptors, although not all, which explains the lack of response to treatment in some patients.

Currently, available somatostatin analogues include octreotide SC, octreotide LAR, and lanreotide. All have a greater potency and longer half-life than physiological somatostatin. Recently, drugs have been developed with an affinity to multiple somatostatin receptors, such as the case of pasireotide that has high affinity for receptors 1, 2, 3, and 5. It is believed that receptors 1 and 3 have major implications in the control of tumor size. In addition, due to the multiplicity of affinity of pasireotide, it may be useful in patients with refractory to octreotide, which acts only on receptors 2 and 5.

Treatment efficacy should be evaluated based on the criteria of disease control; however, the nadir of GH after oral glucose load is not a useful test for treatment with the somatostatin analogue due to the high frequency of discordant results. It may be possible that the suppression directly induced on the hepatocyte by the somatostatin analogue drugs decreases IGF-1 levels or affects the physiological response of GH to oral glucose load. Biochemical control of acromegaly (assessed by GH levels) by using octreotide LAR and lanreotide as postoperative treatment is achieved in 56% and 49% of cases, respectively. On the other hand, normalization of IGF-1 concentration adjusted for age and gender is achieved in 66% and 48% of cases, respectively [33, 34]. The most important factor in these cases is the amount of residual tumor after surgery.

Decrease in tumor size in patients treated with somatostatin analogues was reported in ~30% of patients where a reduction of between 20% and 50% of the tumor mass has been documented. Tumor shrinkage is believed to be due to a deceleration in cell division caused by the drugs, demonstrated by a reduction in Ki67 expression.

#### 15.1.2. Dopamine Agonists

Dopamine agonists are also used in the management of GH-secreting tumors, although results are not as effective. The main advantage is that they are administered orally. The most frequently dopamine agonists used today are long-acting, such as cabergoline and quinagolide, which bind to D2 receptors that are expressed in some pituitary tumors. Bromocriptine has a limited action in these tumors. With cabergoline, decreases of GH levels to <2 *μ*g/L in 46% of patients and also a reduction in IGF-1 level to <300 *μ*g/L in 39% of the cases have been reported [34]. With quinagolide, in 47.8% of patients only a mild reduction in GH level has been achieved (<5 *μ*g/L) [35]. For practical purposes, dopaminergic agonists are recommended in patients with cosecreting GH pituitary and prolactin tumors, especially if GH levels are only slightly elevated. Immunohistochemical analysis of the removed tumor is crucial in order to demonstrate the simultaneous presence of lactotrope and somatotrope tissue because in most cases prolactin blood level is only slightly elevated.

#### 15.1.3. GH Receptor Antagonists

If, after surgery and the use of somatostatin analogues and dopaminergic agonists, control is not achieved, treatment with a GH receptor antagonist is still an option. Pegvisomant is a molecule similar to GH, but mutated at two sites, which provides greater affinity for the receptor. The efficacy of pegvisomant is determined by quantifying IGF-1 serum levels. When these antagonists are indicated, serum levels of GH cannot be used to guide therapeutic response because these drugs generate a positive feedback in the GH by blocking its receptors. This leads to very high levels of this hormone despite an adequate therapy [36].

Reduction of IGF-1 levels has been observed in 90%–97% of patients treated with pegvisomant. However, it was observed that, in up to 6% of the cases, an increase in tumor size was reported. This tumor growth may be explained also due to the lack of negative feedback of GH. In the absence of the union of GH to its receptors, a signal for additional hormone production and cellular proliferation is sent [31].

## 16. Radiotherapy and Radiosurgery

Standard external beam radiotherapy (SEBRT) should almost never be considered as a first or secondary line of treatment in acromegaly. It is indicated when there has been a poor response to surgery and medical management in the presence of a morbid disease or a rapidly growing tumor [37]. It is reported that this therapeutic modality is able to reduce GH and IGF-1 levels in up to 60% of cases but with the disadvantage that the maximum response is achieved after 10–15 years of its application. Radiosurgery (RS) consists of the stereotactic-guided delivery of high doses of ionizing radiation concentrated in a small area [38]. Remission rates at 5 years with the use of RS range from 29%–60%. The main advantages over SEBRT are that it requires less time to achieve this remission [5], has a lower risk of injury to surrounding structures (particularly the visual system), and also represents a lower risk of generating secondary tumors [39]. However, its main limitation is that it is reserved for tumors with a maximum size of 3 cm diameter. The ideal indication for RS is in the treatment of residual tumors (postsurgery) in the CS.

A form of radiation that uses a combination of both modalities is called fractionated stereotactic radiotherapy and is characterized by the concentrated application of a high dose of ionizing radiation but in a fractionated (multiple-session) manner. This fractionation permits the increase in the radiation dose at various points but without increasing side effects in the surrounding tissue. Using this method, tumors larger than indicated for radiosurgery can be treated, but without the side effects of SEBRT.

**Figure 11:**
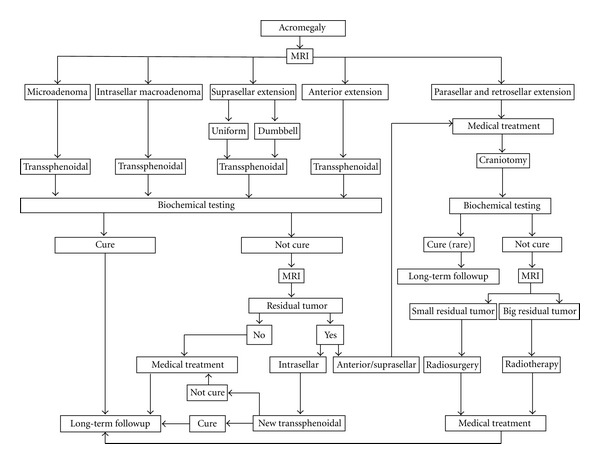
Summary of the most important therapeutic options used today for acromegaly.

A flow diagram is shown on Figure 11 where all these therapeutic options currently used in acromegaly are analyzed. 

## 17. Conclusions

Surgery remains the most effective option to achieve rapid and complete cure for all patients who can have transsphenoidal surgery. Even by advanced endoscopic technique, the chances of remission rate are 55% for those with macroadenoma [40], but the reduction in tumor mass can result in considerable improvement of response after subsequent medications.

There are still reasons for offering craniotomy in selected cases, particularly for huge, extrasellar lesions. Now medical treatment has an increasing importance in the treatment of acromegaly, and first-line medical therapy with somatostatin analogues is being widely used in clinical practice, either prior to surgery or in patients who are poor surgical candidates and in those in whom there is a low probability of a surgical cure. Somatostatin analogues induce clinically significant tumor shrinkage when given as first-line, when this reduction of tumor volume could be helpful in improving the outcome of subsequent surgery or improving the clinical syndrome in patients with unacceptable surgical risk [41]. The treatment of acromegaly is a multidisciplinary treatment strategy involving experienced neurosurgeons, endocrinologists, and radiation oncologists. Consensus guidelines have been described for the optimal management of acromegaly [42]. Nevertheless, an individualized approach for each patient is necessary to determine the most efficacious and cost-effective.
